# Glomus Extradigital Tumor: A Case Report of an Extradigital Glomus Tumor on the Wrist and Comprehensive Review of Glomus Tumors

**DOI:** 10.7759/cureus.38737

**Published:** 2023-05-08

**Authors:** Philip R Cohen

**Affiliations:** 1 Dermatology, University of California, Davis Medical Center, Sacramento, USA

**Keywords:** wrist, tumor, subungual, solitary, multiple, glomus, glomangiosarcoma, glomangioma, extradigital, digital

## Abstract

A glomus tumor is a neoplasm composed of cells from the glomus body which is a contractile neuromyoarterial structure that affects blood pressure and thermoregulation by altering cutaneous blood flow. This cutaneous tumor can be benign or rarely malignant, solitary or multiple, and digital or extradigital. A benign glomus tumor usually presents as a non-familial, solitary, and subungual lesion. Multiple glomus tumors are less common, may be autosomal dominantly inherited, and extradigital. In contrast to a digital glomus tumor that often occurs within the nailbed or fingertip pulp of a young woman, a glomus extradigital tumor (GET) typically appears on the extremity or trunk of an older man. The diagnosis of a glomus tumor may be suspected based on clinical evaluation; it classically presents with a clinical triad of symptoms which includes lesion-associated tenderness, pin-point pressure pain, and cold sensitivity. However, cold-induced exacerbation of pain is frequently absent in extradigital glomus tumors; this may contribute to a delay in establishing the diagnosis of a glomus tumor in these individuals. Radiographic studies can support the diagnosis, but tissue specimen examination is necessary to establish the diagnosis. Resolution of tumor-associated pain is usually achieved following the complete excision of the neoplasm. A woman with a glomus tumor located on her wrist is described; her painful tumor was not sensitive to cold and was misdiagnosed clinically as a possible foreign body reaction to either a wood splinter or glass shard. The diagnosis of an extradigital glomus tumor was established after a microscopic examination of the tissue specimen following an excisional biopsy using a 3-millimeter punch biopsy tool. The neoplasm-related pain ceased and did not recur after the tumor had been completely removed. In conclusion, a glomus tumor is included in the differential diagnosis of a painful cutaneous neoplasm; however, misdiagnosis and/or substantial delay in diagnosis may occur if the tumor is extradigital or it lacks cold sensitivity or both. Therefore, the clinician needs to entertain the possibility of an extradigital glomus tumor when evaluating a patient with a tender, temperature-insensitive skin lesion that is not located on the fingers or toes.

## Introduction

A glomus tumor is usually a benign neoplasm consisting of glomus cells which often presents as a painful dermal tumor [1,2]. It is commonly located beneath the nail plate and referred to as a glomus subungual tumor (GUT) [3-5]. However, albeit less frequently, a glomus tumor may occur at other sites; in this non-subungual location, it is referred to as a glomus extradigital tumor (GET) [6-8].

An extradigital glomus tumor typically presents as a painful dermal nodule that frequently appears on the arm or the leg or the trunk; however, it can occur at any site on the body. The diagnosis of an extradigital glomus tumor can be delayed not only because of its atypical location but also secondary to an absence of tumor-associated cold sensitivity in some of the lesions [9-11]. The features of an extradigital glomus tumor, clinically suspected to be a foreign body, on the right wrist of a 59-year-old woman are described; her symptoms resolved without recurrence following an excisional biopsy using the punch technique that completely removed her tumor.

## Case presentation

A 59-year-old Vietnamese woman presented for evaluation of a tender area beneath the skin on her distal right upper extremity that had been symptomatic for a few months. Although she did not recall experiencing an injury at the location, she was not certain whether the affected area had previously been traumatized. The subcutaneous lesion was very painful when she touched it with the fingers of her left hand. The lesion and surrounding area would also spontaneously become tender. However, the application of cold water to the lesion did not cause pain.

**Figure 1:**
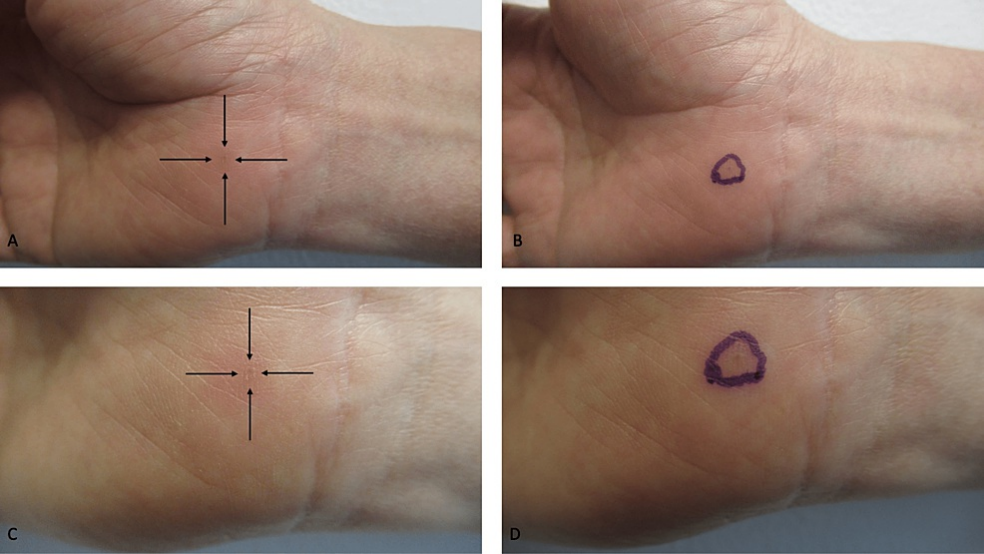
Clinical features of a GET on the distal right upper extremity Distant (A and B) and closer (C and D) views of an extradigital glomus tumor presenting as a 5-millimeter erythematous patch with a 2-millimeter flat-topped papule with a slight central depression on the right flexor wrist of a 59-year-old woman. The red lesion, whose borders are demonstrated by black arrows (A and C) and surrounded by a purple ink oval (B and D), was not only painful when touched but also spontaneously tender.

A cutaneous examination of her right flexor wrist showed a 5-millimeter erythematous patch surrounding a 2-millimeter flat-topped papule which had a slight central depression (Figure 1). Pin-point tenderness was elicited by applying gentle pressure to the lesion. Based on her symptoms and the morphology of the lesion, the primary consideration in the clinical differential diagnosis included a foreign body reaction to either a wooden splinter or a small shard of glass.

**Figure 2:**
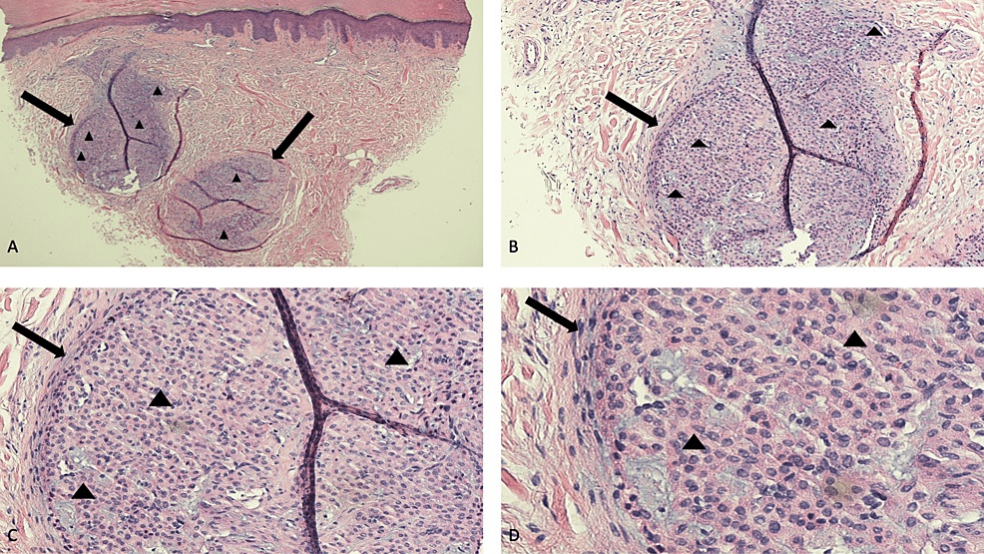
Microscopic features of an extradigital glomus tumor Low (A) and higher (B, C, and D) magnification views of the pathology findings of a GET that was located on the flexor wrist of a 59-year-old woman’s right extremity. The excisional tissue specimen, using a 3-millimeter punch biopsy tool, showed nodular aggregates (black arrows) consisting of solid sheets of basophilic, uniform-appearing, glomus cells (black triangles) in the dermis (hematoxylin and eosin: A, x4; B, x10; C, x20; and D, x40).

An excisional biopsy using a 3-millimeter punch biopsy tool, with the depression area in the center of the biopsy site, was performed; the wound was closed with 5-0 non-absorbable sutures. Microscopic examination of the tissue specimen showed compact hyperkeratosis and mild acanthosis that correlated with the normal thickness of the skin at this location. In the dermis, there were large nodular aggregates of benign-appearing cells. The basophilic tumor consisted of solid sheets of uniform-appearing glomus cells; since the predominant component of the tumor was glomus cells with poor vasculature and sparse smooth muscle, it was classified as a glomus tumor of the solid type. In the sections examined, the neoplasm appeared to be completely removed (Figure 2).

Correlation of the clinical symptoms, lesion morphology, and pathologic findings established a diagnosis of an extradigital glomus tumor. Two weeks after the biopsy, she returned for suture removal. Examination at the follow-up visit showed that the biopsy site had completely healed and the sutures were removed; in addition, the lesion site was no longer painful. There has been no recurrence of symptoms during the subsequent 20 months.

## Discussion

The initial clinical description of a glomus tumor, as a painful subcutaneous tubercle, was provided by Wood in 1812. Subsequently, in 1878, Kolaczek reported on their typical subungual location. However, it was not until 1924 that Barre and Masson described the microscopic features of the glomus tumor and established its origin in the glomus body [1,2,5,11].

Glomus tumors represent 2% of all soft tissue tumors. The World Health Organization recently classified glomus tumors into three types: benign, intermediate glomangiomatosis, and malignant [12].

Glomangiomatosis is also referred to as a diffuse glomus tumor. It represents only 5% of glomus tumors; a recent review only includes 17 patients. A diffuse glomus tumor usually presents in young people as a solitary lesion on the distal extremities [12].

Malignant glomus tumors are also referred to as glomangiosarcoma. They either develop de novo or rarely from a prior benign tumor. In a retrospective study of 50 patients with one glomus tumor (48 patients) or multiple glomus tumors (two patients), only one patient had a glomangiosarcoma located on the upper extremity [2]. Indeed, less than 50 reports of cutaneous malignant glomus tumors have been reported [2,13,14].

The glomus body, located in the reticular dermis, is a contractile neuromyoarterial structure. Glomus cells surround the arterial end (referred to as the Sucquet-Hoyer canal) of the glomus body. As a specialized form of arteriovenous anastomosis, the glomus body can change the blood flow within the skin and thereby affect not only blood pressure control but also thermoregulation [1-3,5-9,11,13,15].

Glomus bodies are most numerous in the distal fingers and toes; this accounts for the prevalence of glomus tumors in the subungual location of the digits. Since glomus bodies can be found throughout the entire body, extradigital glomus tumors of the skin have also been observed. In addition, an atypical or ectopic glomus tumor refers to the occurrence of a lesion where glomus bodies do not physiologically exist, such as visceral organs [1-3,5-9,11,13,15].

The pathogenesis of a glomus tumor remains to be established. Several factors have been observed in patients with a glomus tumor that may influence their etiology. These include not only gender and a prior history of trauma to the site but also onset age and familial occurrence. For example, subungual tumors are more common in younger women, extradigital tumors are more frequent in older men, and multiple glomus tumors have been observed not only to be present at birth or early infancy but also to occur with an autosomal dominant pattern of inheritance [3,5,8,10-12,16].

A benign glomus tumor occurs as either a solitary mass or multiple lesions (Table 1) [1,2,5,6,12,16-18]. The solitary tumor is the more common. It is typically presented as an acquired, painful, reddish-blue, firm lesion in a 30- to 40-year-old woman. It is often subungual; however, it can also be extradigital.

**Table 1:** Characteristics of patients with solitary and multiple glomus tumors cm, centimeters; mm, millimeters; p, short arm of chromosome

Characteristic	Solitary glomus tumor	Multiple glomus tumors
Nomenclature		
Name	Glomus tumor	Glomangioma, glomuvenous malformation
Epidemiology		
Occurrence	Common	Rare
Inheritance	Acquired, rarely familial	Familial (autosomal dominant), non-familial
Chromosome association	Not applicable	1p21-22
Gender	Female greater than male	Male greater than female
Onset age	Adulthood: fourth decade (women) to fifth decade (men)	Congenital, infancy, early childhood, and early adulthood
Malignant tumor	Rare	None reported
Symptoms		
Pain	Majority	Minority
Clinical features		
Number of lesions	One	Two or more
Size of lesion	Two mm - one to two cm	Two mm - more than four cm
Color	Reddish-blue	Blue
Palpation consistency	Firm	Soft
Distribution	Localized	Localized (regional), generalized (disseminated), or segmental
Location	Subungual or extradigital (such as the distal limbs)	Extradigital (extremities or trunk)
Pathology		
Capsule	Fibrous	Non-encapsulated
Blood vessels	Small cavities	Irregular, large, and ectatic
Glomus cells (two to three layers)	High number	Few

Multiple glomus tumors are referred to as a glomangioma or a glomuvenous malformation (Table 1) [1,2,5,6,12,16-18]. A glomangioma occurs less frequently than a solitary glomus tumor and can be inherited in an autosomal dominant manner. Glomulin (GLMN), a phosphorylated protein that is a member of a Skp1-Culin-F-box-like complex, is encoded by the GLMN gene which is located on the short arm of chromosome 1 (1p21-22). GLMN is essential for the normal development of the vasculature and glomangioma has been associated with a mutation that results in the inactivation of the GLMN gene [12].

Multiple glomus tumors are more common in men (Table 1) [1,2,5,6,12,16-18]. They appear earlier than a solitary glomus tumor, either as congenital lesions or tumors appearing in infancy, early childhood, or early adulthood. They often present as painless blue extradigital lesions on the extremities or trunk. Glomuvenous malformations can be localized to a region of the body, or segmental, or have a generalized distribution with widely disseminated tumors.

A glomus tumor can be red to pink to blue to purple in appearance (Table 2) [1-3,5,6,15]. It is usually smaller than one centimeter. However, the lesion can be only two millimeters in size and is difficult to localize on clinical examination.

**Table 2:** Clinical presentation of a suspected glomus tumor

Presentation	Comment	Ref
Morphology	Lesions appear bluish to pinkish-red and are usually less than one centimeter in diameter.	[1,13]
Symptoms	The classic triad of clinical symptoms includes pain, pin-point tenderness, and cold sensitivity. However, lesion-associated pain may be absent in individuals with multiple tumors. In addition, sensitivity to cold may be absent in patients with subungual tumors and more frequently is not present in persons with extradigital tumors.	[2,3,6]
Dermoscopy	Subungual glomus tumors can show linear vascular structures on nail plate dermoscopy; however, investigators have observed four dermoscopic patterns (in order of greatest to least sensitivity): (1) a structureless purplish/red subungual spot with or without vessels; (2) a structureless purplish/red subungual spot and longitudinal erythronychia; (3) a structureless proximal purple/red subungual spot, longitudinal erythronychia, and a V-distal notch (this dermoscopic pattern was only found in glomus tumors located on the nail matrix); and (4) a structureless subungual nail bed spot and distal onycholysis (this dermoscopic pattern most commonly occurred with nail bed tumors). Extradigital glomus tumors show a homogenous white structure (described as a structureless purple-white to reddish-white homogenous area) and peripheral telangiectasias (which have also been described as linear unfocused vessels).	[5,15]
Transillumination	This can be helpful for not only the diagnosis but also the location of subungual tumors. The tumor appears as a red opaque image when light is passed through the pad of the finger.	[1,3]

There are several painful dermal neoplasms; the glomus tumor is included in the list of these lesions [19]. In addition to pain, pin-point tenderness and cold sensitivity are the other features that comprise the classic triad of clinical symptoms associated with a glomus tumor. However, cold sensitivity is often absent in an extradigital glomus tumor. Indeed, the extradigital glomus tumor on the wrist of the woman in this report only presented as a tender lesion that exhibited pin-point pain but did not have any cold sensitivity.

Dermoscopy and transillumination may be helpful in the clinical evaluation of an individual in whom a glomus tumor is suspected. Four dermoscopic patterns have been defined in patients with a subungual glomus tumor (Table 2) [1-3,5,6,15]. A homogenous white structure and peripheral telangiectasias have been observed during the dermoscopy of an extradigital glomus tumor [5,15]. A red opaque image can be visualized when light is passed through the finger pad of a digit with a subungual glomus tumor; hence, in individuals with a subungual glomus tumor, transillumination can be useful for both diagnosing and localizing the lesion [1,3].

A glomus tumor of the skin can either be subungual (a GUT) or extradigital (a GET). The features of these tumors are summarized in Table 3 [1-5,7-11,18]. A subungual glomus tumor is more frequently occurring, more common in women, and has a younger onset age than an extradigital glomus tumor. It typically presents with pain, pin-point tenderness, and cold sensitivity; therefore, in individuals with a subungual glomus tumor, a clinical misdiagnosis is less common, and the duration of time between symptom onset and diagnosis is usually shorter than that observed in patients with an extradigital glomus tumor.

**Table 3:** Characteristics of patients with subungual and extradigital glomus tumors cm, centimeter; GET, glomus extradigital tumor; GUT, glomus subungual tumor; mm, millimeter; <, less than; >, greater than; % percent ^a^In a study of individuals with a glomus tumor, 33 of 50 patients had a subungual glomus tumor [2]. ^b^In a study of individuals with a glomus tumor, 17 of 50 patients had an extradigital glomus tumor [2]. ^c^Prior trauma to the site of a subungual glomus tumor was observed in 14 of 73 (19%) of patients in one study [10]; other investigators cite an incidence of 75% of subungual glomus tumors being preceded by trauma at the tumor location [3,5]. ^d^Prior trauma to the site of an extradigital glomus tumor was observed in 11 of 56 (20%) of patients in one study [11]; other researchers cite an incidence ranging from 20% to 30% of extradigital glomus tumors being preceded by trauma at the tumor location [4]. ^e^Subungual glomus tumors are more frequent in women than in men. Studies of patients with a subungual glomus tumor have observed the female-to-male ratio to range from 21:16 [4] to 4:1 [2] to 5:1 [1]. ^f^Extradigital glomus tumors are usually more common in men than in women. Studies of patients with an extradigital glomus tumor have observed the male-to-female ratio to range from 3:2 [1] to 4:1 [9] to 4.1:1 [11]; however, one study noted the male-to-female ratio to be 1:3 [2]. ^g^One study of patients with a subungual glomus tumor noted a delay of diagnosis ranging from two to 12 years (mean, 3.8 years) [4]; other researchers cited a delay of 0.5 to 12.5 years [1]. ^h^One study of patients with an extradigital glomus tumor noted a delay of diagnosis ranging from five to 25 years (median, 16 years) [1]; other investigators cited a delay of five to 20 years [11].

Characteristic	Subungual glomus tumor	Extradigital glomus tumor
Nomenclature		
Name	Digital glomus tumor, GUT	Slledsdivesn glomus tumor, GET
Epidemiology		
Occurrence	Frequent	Less common
Incidence	66% to 75%^a^	25% to 34%^b^
History of prior trauma to site	19%^c^	20% to 30%^d^
Gender	Female greater than male^e^	Male greater than female^f^
Onset age	Third to fifth decade	Fourth to sixth decade
Clinical misdiagnosis	Less common	Frequent
Delayed diagnosis duration	0.5 to 12.5 years^g^	Five to 25 years^h^
Symptoms		
Painful	Yes	Yes
Cold exacerbates pain	Common	Less frequent
Clinical features		
Size	Smaller (<10 mm)	Larger (>one cm)
Color	Bluish or reddish	Blue, purple (violaceous), reddish-blue, red-purple
Consistency	Firm	Hard
Shape	Oval	Nodule
Site	Subungual, finger pulp (less common)	Extremity, trunk
Location	Nail bed (dermis) or pulp of the finger	Dermis or subcutaneous
Associated nail deformity	Yes: elevated, deformed, or discolored	None

A digital glomus tumor is most frequently located in the subungual nail bed of the finger or thumb. However, the tumor can occur in the pulp at the tip of the digit. The latter site has been considered rare; it has been described in single case reports or occurred in only one of 11 patients in a retrospective study [1,5]. However, other researchers have observed a glomus tumor of the digit with a greater frequency, being present in either four of 37 individuals (11%) with a glomus tumor of the fingertip or 18 of 73 patients (25%) with a glomus tumor involving either the upper limb or hand [4,10].

A subungual glomus tumor is usually a small lesion, typically less than one centimeter in size. It is a bluish or reddish firm, oval papule. Frequently, the color or the shape or both of the overlying nail plate has a clinically observable alteration [4].

An extradigital glomus tumor occurs less commonly than a subungual glomus tumor, is usually more frequent in men, and has an older onset age than a subungual tumor (Table 3) [1-5,7-11,18]. They are usually painful; however, tumor tenderness is less frequently exacerbated by cold. Therefore, the possibility of a glomus tumor is often not initially considered, and there can be a long delay between the onset of symptoms and establishing the diagnosis.

An extradigital glomus tumor is typically located on either the extremities or trunk. Similar to a subungual glomus tumor, an extradigital glomus tumor varies in color from blue to red to purple. However, an extradigital glomus tumor is usually larger than a subungual glomus tumor, often being greater than one centimeter in size [1-11,18].

The wrist is a rare location for an extradigital glomus tumor; including the woman in this report, at least 22 patients with a glomus tumor at this site have been described (Table 4) [1,10,11,14,20]. Similar to other extradigital glomus tumors, those located on the wrist occurred more commonly in older men than younger women. Although all tumors were tender, the pain was not exacerbated by cold in four patients; this may have contributed to the delay in diagnosis after the pain onset that was observed. Some of the patients with glomus tumors of the wrist had lesions that were larger, intravascular, and/or malignant.

**Table 4:** Characteristics of patients with an extradigital glomus tumor of the wrist ^a^Two studies evaluated the incidence of an extradigital glomus tumor of the wrist within a study group of patients with a glomus tumor; the incidence ranged from 2.7% (two of 73 patients) [10] to 9.1% (one of 11 patients) [1]. ^b^Three studies evaluated the incidence of an extradigital glomus tumor of the wrist within a study group of patients with an extradigital glomus tumor; the incidence ranged from 7.1% (four of 56 patients) [11] to 10% (two of 20 patients) [10] to 20% (one of five patients) [1].

Characteristic	Comments
Epidemiology	
Number of patients	An extradigital glomus tumor of the wrist has been reported in at least 22 patients.
Occurrence	Extradigital glomus tumor of the wrist is rare.
Incidence	The incidence of an extradigital glomus tumor of the wrist ranged from 2.7% to 9.1% of all glomus tumors and 7.1% to 20% of extradigital glomus tumors.^a,b^
History of prior trauma to site	Trauma to the tumor site from either a wooden splinter or a small shard of glass, glass debris (as a foreign body), or a contusion were each described in one patient. However, five patients stated that there was no prior trauma to the tumor site.
Gender	There were 12 men and four women with an extradigital glomus tumor of the wrist; the gender was not described for six patients. The ratio of men to women was 3:1.
Onset age	The onset age for all patients ranged from 29 to 79 years (median, 63 years). The onset age for men ranged from 33 to 79 years (median, 63 years). The onset age for women ranged from 29 to 59 years (median, 45 years).
Clinical misdiagnosis	A misdiagnosis was reported in three patients: extensor tendonitis, fluid collection, or foreign body reaction to a wood splinter or glass shard.
Delayed diagnosis duration	The duration of pain prior to establishing the diagnosis of extradigital glomus tumor ranged from three months to 20 years (median, four years).
Symptoms	
Pain	All the patients experienced tumor-associated pain.
Cold does not exacerbate pain	Four patients (three women and one man) stated that their tumor-associated pain was not exacerbated by cold.
Clinical features	
Size	The greatest tumor dimension was either less than one centimeter (three patients), between one to two centimeters (five patients), between two to three centimeters (five patients), or three or more centimeters (two patients).
Consistency	The tumor consistency to palpation was either hard (two patients) or soft (two patients). The tumor consistency was not described for the other patients; tumor size was not stated for the other patients.
Pathology	
Intravascular	The glomus tumor originated in the wall of a vein and protruded into the lumen of that vessel in two patients.
Malignant	A cutaneous malignant glomus tumor, either adjacent to a benign glomus tumor or alone, was observed in two patients.

Approximately one-fifth of individuals with either a subungual or an extradigital glomus tumor have a prior history of trauma to the lesion site [10,11]. Investigators summarized the features of eight patients who had an extradigital glomus tumor of their upper or lower extremities that was preceded by a history of local trauma. They found that injury occurred between two weeks to 21 years prior to the diagnosis of the tumor and that the types of injury included prior surgery (two patients), ruptured tendon repair (two patients), puncture from either a knife or tree (two patients), glass injury (one patient), and low-level trauma (one patient) [8].

The clinician may not suspect the diagnosis of a glomus tumor during the initial evaluation of the patient. The physical examination may be unremarkable, particularly in an individual with a tumor that is situated deep in the pulp of the fingertip or a person with a very small extradigital lesion. In addition, some patients may have nonspecific tumor-related symptoms, and many individuals, especially those with an extradigital glomus tumor, do not present with all three of the symptoms of the classic triad: pain, pin-point tenderness, and cold sensitivity [4,9].

Several investigators have demonstrated that establishing the diagnosis of a glomus tumor is often delayed by several months to many years after the onset of symptoms [5-8,12]. In a study of 11 patients with a glomus tumor, the diagnosis was established between 0.5 to 25 years (median, 15 years) after symptom onset; the duration of time between symptom onset and diagnosis ranged from 0.5 to 22 years (median, 12.5 years) for the patients with a digital lesion and from five to 25 years (median, 16 years) for the patients with an extradigital tumor [1]. In another investigation of 73 patients with either a digital or an extradigital glomus tumor of the upper limb and hand, the mean time to diagnosis after symptom onset was six years, and the incidence of missed diagnosis was 31% (23 patients) [10].

The diagnosis delay after symptom onset was also evaluated in studies that included individuals with only a digital or an extradigital glomus tumor. In an investigation of 37 patients with a glomus tumor of the fingertip, the delay of diagnosis post-symptom onset ranged from two to 27 years (mean, 3.8 years) [4]. However, a longer mean duration of diagnosis delay, 7.4 years, was observed in 44 patients with an extradigital glomus tumor [11].

The delay in the diagnosis of a glomus tumor also influenced the mental health of some of the patients. Many of the individuals experienced a prolonged duration of excruciating chronic pain. Indeed, especially for patients with very small tumors, the pain was often discordant with the size of the tumor. In addition, particularly in the setting of an extradigital glomus tumor, psychiatric misdiagnoses were assigned to some of those individuals whose lesion could not be clinically appreciated since it was either too small or too deeply located [8,11].

Patients with a glomus tumor typically visit numerous physicians and healthcare professionals in an effort to discover the etiology of their painful lesion [4]. Indeed, one group of investigators commented that individuals with a glomus tumor experienced 2.5 consultations with a specialist prior to establishing the diagnosis [8]. The spectrum of clinicians who evaluated glomus tumor patients was extensive; in a study of 54 patients with an extradigital glomus tumor, encounters with the following individuals were recorded: acupuncturist, chiropractor, dermatologist, neurologist, neurosurgeon, orthopedist, primary care physician, and rheumatologist [11].

The clinical differential diagnosis of a subungual glomus tumor includes not only benign solid tumors, benign cystic tumors, and malignant tumors but also vascular lesions, adnexal lesions, and melanocytic lesions (Table 5) [4,15].

**Table 5:** Clinical differential diagnosis of a subungual glomus tumor

Condition	References
Angioleiomyoma	[4]
Blue nevus	[4]
Blue rubber bleb nevus syndrome	[4]
Chondroma	[4]
Eccrine spiradenoma	[4]
Glomus tumor	[4]
Hemangioma	[4]
Kaposi sarcoma	[4]
Keratoacanthoma	[4]
Maffucci syndrome	[4]
Malignant melanoma	[4]
Mucous cyst	[4,15]
Neurilemmoma	[4]
Onychopapilloma	[15]
Pacinian corpuscle (hyperplastic)	[4]
Pyogenic granuloma	[4]
Squamous cell carcinoma	[4]
Subungual exostosis	[4]
Venous malformation	[4]

Several of these conditions are also in the clinical differential diagnosis of an extradigital glomus tumor (Table 6) [2,3,5,6,8,9,11,15]. However, a retrospective study of 56 extradigital glomus tumors noted that hemangioma, neuroma, and neurofibroma were the most common misdiagnoses [11].

**Table 6:** Clinical differential diagnosis of an extradigital glomus tumor CR, current report; NOS, not otherwise specified

Condition	References
Abdominal pain (chronic)	[9]
Angioma	[11]
Arteriovenous malformation	[9,11]
Blue rubber bleb nevus syndrome	[3]
Cavernous hemangioma	[3]
Chondroma	[8]
Chondromalacia	[8]
Complex regional pain syndrome	[8]
Cyst, infected	[9]
Dermatofibroma (aneurysmal)	[15]
Eccrine spiradenoma	[3,5,8,15]
Epicondylitis	[8]
Foreign body granuloma (reaction)	[8], CR
Ganglion cyst	[8]
Glomus tumor	[8,11]
Gouty arthritis	[3,5]
Hemangioma	[3,8,11,15]
Kaposi sarcoma	[15]
Leiomyoma	[3,5,15]
Lipoma	[11]
Malignant melanoma (amelanotic and NOS)	[5,15]
Medial plica syndrome	[8]
Neuritis	[8]
Neurofibroma	[6,8,11]
Neuroma (NOS and traumatic)	[2,3,6,8,9,11,15]
Nevus (intradermal)	[11]
Peripheral nerve sheath tumor	[8]
Peripheral neuropathy	[8]
Pyogenic granuloma	[15]
Radiculopathy	[8]
Sarcoma	[5,8]
Schwannoma	[6,8]
Tendonitis	[8]
Tenosynovitis	[9]

A possible history of trauma or injury to the wrist of the woman in this report was considered to have occurred. Therefore, the diagnosis of a foreign body reaction to either a wood splinter or glass shard was suspected. The possibility of an extradigital glomus tumor was not entertained; hence, additional clinical or radiographic evaluations were not performed prior to the excisional biopsy using a punch biopsy tool which not only established the diagnosis but also resolved all the glomus tumor-associated symptoms.

Several clinical tests can be used to evaluate individuals in whom a glomus tumor is suspected (Table 7) [1-3,6,8]. The examinations focus on exacerbating or relieving tumor-associated pain. The cold sensitivity test is performed by exposure to cold weather or applying either cold water or a cold object to the area where the tumor is located; increased pain at the site is a positive test result [2,3,8].

**Table 7:** Clinical tests that can be performed on an individual in whom a glomus tumor is suspected

Test	Comment	References
Hildreth test	A positive test result is demonstrated by the relief of pain when ischemia is produced by applying a tourniquet on the affected limb proximal to the tumor; for example, this can be accomplished by inflating a cuff around the arm.	[1-3,6]
Love (pin) test	A positive test result is demonstrated by the occurrence of localized pain after pin-point pressure is applied to the area where the tumor is suspected; the tumor is usually located at the site where the pain is most severe.	[2,3,6]
Modified Love test	A positive test result is demonstrated by either a decrease in pain or the absence of pain, caused by the temporary ischemia that results from a restricted blood supply to the area when the Love test is performed after a tourniquet has been applied proximally to the affected limb.	[2]
Cold sensitivity test	A positive test result is demonstrated by the individual experiencing increased pain after the tumor site is soaked in cold or icy water. Similarly, tumor-related symptoms are exacerbated in cold weather or when cold objects are held against the tumor.	[2,3,8]

The Hildreth test involves the application of a tourniquet to the limb on which the tumor is located. Tourniquet-induced ischemia results in pain relief of the lesion. Therefore, diminished tenderness of the tumor is a positive test result [1-3,6].

The Love test is performed by applying pin-point pressure to the area of the lesion. The glomus tumor is located at the site where the most severe pain is elicited. In contrast, the modified Love test involves applying pin-point pressure after a tourniquet has been applied to the tumor-affected limb; absence or decreased pain is a positive test result [2,3,6].

Radiologic evaluation of a lesion suspected to be a glomus tumor may aid in the location of the lesion (Table 8) [ 2,3,6,8-11]. A roentgenogram may demonstrate cortical thinning or erosion of the distal phalanx associated with a subungual tumor [2,3,11]. Computerized tomography can also be helpful but is less frequently performed [11].

**Table 8:** Radiographic modalities that can be utilized in the evaluation of a suspected glomus tumor

Modality	Comment	References
Roentgenogram	An erosion or cortical thinning of the bone of the distal phalanx, adjacent to the glomus tumor, may be observed on the roentgenogram of a patient with a subungual lesion.	[2,3,11]
Ultrasound: routine Doppler sonography	The routine Doppler ultrasound can show the size (by detecting tumors as small as two millimeters in diameter), location, and shape of the tumor. Doppler ultrasound is not only good for assessing blood flow around the lesion but also for differentiating a solid lesion (such as a glomus tumor) from a cystic lesion.	[2,3,6,9]
Ultrasound: color Doppler sonography	The color Doppler ultrasound can differentiate glomus tumor from other soft tissue tumors; the glomus tumor hypervascularity is demonstrated by visualizing the arterial patterns of the tumor. The glomus tumor appears as a well-circumscribed subcutaneous mass that is hypervascularized and hypoechoic.	[2,8]
Computerized tomography	Computerized tomography can detect extradigital tumors.	[11]
Magnetic resonance imaging	Magnetic resonance imaging can detect tumors as small as two millimeters in diameter; it can also be useful to exclude other tumors in the differential diagnosis of a glomus tumor. T1-weighted images show low signal intensity. T1-weighted images after gadolinium injection show enhancement. T2-weighted images show high signal intensity. There are important caveats to consider: (1) the absence of a glomus tumor cannot be established solely based on a negative magnetic resonance imaging scan, and (2) other solid tumors and cysts can present with the same classic magnetic resonance imaging findings (a dark lesion on T1 and a bright lesion on T2) as a glomus tumor.	[2,3,6,8-11]

Sonography and magnetic resonance imaging can also be performed when assessing whether a lesion is a glomus tumor; both have a similarly high sensitivity. However, ultrasound may be the preferred modality for evaluation since magnetic resonance imaging only has a specificity of about 50%, is more time-consuming to perform, and is more expensive. In addition, a glomus tumor may be present even when the magnetic resonance image is determined to be negative for the tumor [2,3,6,8-11].

Although the diagnosis of a glomus tumor can be suspected based on clinical features and radiographic examination, a microscopic evaluation of the lesion is required to establish the diagnosis. Three histologic types of glomus tumors have been observed: solid, glomangioma, and glomangiomyoma (Table 9) [2,5-7,11].

**Table 9:** Histologic types of glomus tumors ^a^Also referred to as solid tumor proper.

Histologic type	Synonym	Glomus cells	Blood vessels	Smooth muscle cells
Solid	Solid^a^	Predominant	Poor	Sparse
Glomangioma	Vascular	Sparse	Predominant	Sparse
Glomangiomyoma	Myxoid	Sparse	Prevalent	Prevalent

These classifications can be differentiated on tumor tissue that has been stained with hematoxylin and eosin (Table 10) [1,2,6-8,12-14]. They are based on the number of glomus cells, blood vessels, and smooth muscle cells that are present in the tumor [2,5-7,11].

**Table 10:** Pathologic features of glomus tumors Bcl-2, B-cell lymphoma 2; CD31, cluster of differentiation 31; CD34, cluster of differentiation 34; EMA, epithelial membrane antibody; HMB-45, human melanoma black-45; MSA, muscle-specific actin; p63, tumor protein 63; S100, an acid, calcium-binding, protein characterized by its partial solubility in saturated (100%) ammonium sulfate; SMA, smooth muscle actin; Sox 10, SRY (sex-determining region y)-related HMG (high mobility group) box 10.

Stain	Comment	References
Hematoxylin and eosin	Various amounts of glomus cells, blood vessels, and smooth muscle cells characterize the histologic types of glomus tumors. In the solid (glomus tumor proper) type, solid sheets of glomus tumor cells are present in the dermis. In the vascular (glomangioma) type, the glomus tumor cells surround the blood vessels. In the myxoid (glomangiomyoma ), sparse glomus tumor cells are present in addition to prevalent blood vessels and smooth muscle cells.	[2,6,7]
Immunoperoxidase staining positive	Bcl-2, MSA, SMA, type IV collagen, and vimentin.	[1,2,8,12,13]
Immunoperoxidase staining negative	CD31, chromogranin, cytokeratin, desmin, EMA, factor VIII, HMB-45, p63, S100, Sox10, and synaptophysin.	[1,8,12,14]
Immunoperoxidase stain variable	Caldesmon: weakly positive, calponin, slightly to strongly positive, and CD34: the endothelial cells of vessels in the lesion are positive while the tumor cells are either partially positive, positive, or negative.	[1,8,12,13]

Immunoperoxidase staining may be necessary to either confirm the diagnosis or to rule out other, similar appearing, tumors (Table 10) [1,2,6-8,12-14]. A glomus tumor uniformly expresses smooth muscle actin and vimentin [1,2,8,12,13]. Yet, the tumor does not stain with antibodies directed toward cytokeratin or melanocytic markers such as acid, calcium-binding, protein characterized by its partial solubility in saturated (100%) ammonium sulfate (S100), human melanoma black-45 (HMB-45), and sex-determining region y (SRY)-related high mobility group box 10 (Sox 10) [1,8,12,14].

The pathologic differential diagnosis of a glomus tumor predominantly includes vascular and smooth muscle tumors (Table 11) [2,11,12]. In addition, the malignant variant of the tumor may need to be excluded. In a retrospective study of extradigital glomus tumors observed during a 20-year period, nine of the initial 137 lesions originally classified as a glomus tumor were thought to have been misdiagnosed; the revised diagnoses for these lesions included hemangioma (six tumors), intradermal nevus (one tumor), myofibroma (one tumor), and myopericytoma (one tumor) [11].

**Table 11:** Pathologic differential diagnosis of a glomus tumor

Condition	References
Epithelioid angiomyolipoma	[12]
Hemangioma	[2,11]
Malignant glomus tumor	[12]
Myofibroma	[11]
Myopericytoma	[11]
Nevus (intradermal)	[11]
Paraganglioma	[12]

Glomus tumor is included in the conditions characterized by tender cutaneous neoplasms [19]. Pain associated with the lesion not only occurs spontaneously but also can be elicited by pin-point pressure or exposure to cold. Although the definitive pathogenesis of tumor-related pain remains to be established, several hypotheses have been proposed [3,5,8].

The glomus tumor is typically surrounded by a connective tissue capsule in the dermis. The capsule can be sensitive to pressure. Hence, this may account for the pain observed after the application of pin-point pressure to the lesion [3,5].

Pressure on nerves associated with a glomus tumor can also result in pain. Specifically, non-myelinated nerve fibers may occasionally penetrate the glomus tumor. Pain can result from excessive dominance over the nerve [3,5].

Mast cells have also been observed in the dermis surrounding a glomus tumor. They can release several substances to which pressure receptors and thermal receptors are sensitive. These include heparin, histamine, and 5-hydroxytryptamine [3,5].

Substance P and transient receptor potential cation channel subfamily V member 1 (TRPV1) are molecular mediators for pain and cold sensitivity. High expression of these mediators has been observed in glomus tumors. Hence, the lesion-associated symptoms in a patient with a glomus tumor may be secondary to the presence of these mediators [8].

Difficulty establishing the diagnosis of a glomus tumor has often resulted not only in a delay of tumor-directed therapy but also in the non-effective treatment of the condition [3,4,11]. Some of the non-effective modalities used by chiropractors included acupuncture and manipulation [11]. In a retrospective review of 37 patients with a glomus tumor in the thumb or fingertips, several inappropriate approaches that were advised included amputation, carpal tunnel release, cortisone injections, laminectomy, physiotherapy, radiotherapy, sympathectomy, ulnar nerve decompression, and vasodilator therapy [4].

Excision is the definitive treatment for a glomus tumor, whether it is a GUT or a GET. Persistent or recurrent pain after glomus tumor excision may be an indicator of a residual neoplasm or a new tumor; it can occur in the postoperative period or later [2]. If symptoms persist more than three months after surgery, repeat imaging or repeat exploration or both should be considered [3].

Early recurrence of pain is suggestive of an incompletely excised tumor. This more frequently occurs after the attempted removal of a subungual glomus tumor, depending on the surgical approach that has been used. Alternatively, the suspected tumor may have been completely removed, but a second tumor may have also been concurrently present that had not been diagnosed and therefore not excised [3,4,10,11].

Late recurrence of pain, perhaps two or three years later, introduces the possibility of the development of a new glomus tumor occurring in the same area as the prior lesion [3]. Finally, a later onset of tenderness at the excision site may indicate the development of a postoperative neuroma [4,10,11]. This has more commonly been observed after the excision of a glomus tumor located on the fingertip [10].

The patient in this report had localized tenderness for approximately three months. Although a glomus tumor is included in the differential diagnosis for painful tumors of the skin, the possibility of an extradigital glomus tumor was not suspected [19]. However, as part of the evaluation at her initial visit, an excisional biopsy using a punch biopsy tool, which narrowly removed the entire extradigital glomus tumor, was performed. After the procedure, all the tumor-associated tenderness completely resolved, and the pain did not recur.

## Conclusions

A benign glomus tumor can be non-familial or autosomal dominantly inherited, solitary or multiple, and digital or extradigital. The most frequent presentation is a non-inherited, painful, solitary subungual lesion in a young woman. Tumor-associated tenderness, pin-point pressure pain, and cold sensitivity are glomus tumor-related clinical symptoms. However, sensitivity to cold may be absent in an extradigital tumor, thereby resulting in an incorrect and delayed diagnosis; therefore, a microscopic evaluation of the lesion is necessary to establish the diagnosis. Complete tumor removal usually results in immediate and sustained elimination of the neoplasm-related pain. A woman with an extradigital glomus tumor is reported; based on her clinical history and symptoms, the tender, cold insensitive, neoplasm on her wrist was misdiagnosed as a trauma-related reaction to a foreign body. After a biopsy that removed the entire lesion was performed, the diagnosis of an extradigital glomus tumor of the wrist was established, and she experienced complete and sustained resolution of her tumor-associated pain. In conclusion, when evaluating a patient with a tender, temperature-insensitive skin lesion that is not located on the fingers or toes, the clinician needs to entertain the possibility of an extradigital glomus tumor.

## References

[1] Abou Jaoude JF, Roula Farah A, Sargi Z, Khairallah S, Fakih C (2000). Glomus tumors: report on eleven cases and a review of the literature. Chir Main.

[2] Chou T, Pan SC, Shieh SJ, Lee JW, Chiu HY, Ho CL (2016). Glomus tumor: twenty-year experience and literature review. Ann Plast Surg.

[3] Morey VM, Garg B, Kotwal PP (2016). Glomus tumours of the hand: review of literature. J Clin Orthop Trauma.

[4] Santoshi JA, Kori VK, Khurana U (2019). Glomus tumor of the fingertips: a frequently missed diagnosis. J Family Med Prim Care.

[5] Senhaji G, Gallouj S, El Jouari O, Lamouaffaq A, Rimani M, Mernissi FZ (2018). Rare tumor in unusual location - glomus tumor of the finger pulp (clinical and dermoscopic features): a case report. J Med Case Rep.

[6] Alyaseen HN, Al Ghadeer HA, Al-Ghanim ME, Aljawad HH, Cordoba CR (2021). Extradigital glomangioma of the cutaneous chest wall. Cureus.

[7] Bhat MR, George AA, Pinto AC, Sukumar D, Lyngdoh RH (2012). Violaceous painful nodule of the leg in an Indian male patient. Indian J Dermatol Venereol Leprol.

[8] Ajala RT, Lyon KA, Lyon PR, Harris FS (2021). Extradigital glomus tumor mimics an intrinsic nerve tumor in a trauma patient: case report and literature review. Cureus.

[9] Temiz G, Şirinoğlu H, Demirel H, Yeşiloğlu N, Sarıcı M, Filinte GT (2016). Extradigital glomus tumor revisited: painful subcutaneous nodules located in various parts of the body. Indian J Dermatol.

[10] Jabir S, Rodrigo T, Petkar M, Iwuagwu F (2022). Glomus tumours of the upper limb and hand. A clinicopathological review of cases over two decades. J Hand Surg Eur Vol.

[11] Schiefer TK, Parker WL, Anakwenze OA, Amadio PC, Inwards CY, Spinner RJ (2006). Extradigital glomus tumors: a 20-year experience. Mayo Clin Proc.

[12] Wu RC, Gao YH, Sun WW, Zhang XY, Zhang SP (2022). Glomangiomatosis - immunohistochemical study: a case report. World J Clin Cases.

[13] Kitz R, Leung O, Royer M (2023). A rapidly growing cutaneous malignant glomus tumor with a CCND3 mutation. J Cutan Pathol.

[14] Larsen N, Pavlidakey P, Harada S, Prieto-Granada CN (2023). Cutaneous malignant glomus tumor with an MIR143(CARMN)::NOTCH2 fusion. J Cutan Pathol.

[15] Álvarez-Salafranca M, Bañuls J, Thomas L (2022). Dermoscopy of glomus tumour: a cross-sectional study of 86 cases. J Eur Acad Dermatol Venereol.

[16] Jelinek JE (1982). Aspects of heredity, syndromic associations, and course of conditions in which cutaneous lesions occur solitarily or in multiplicity. J Am Acad Dermatol.

[17] Naversen DN, Worland RG, Burket JM (1986). Giant glomangioma. J Am Acad Dermatol.

[18] Blume-Peytavi U, Adler YD, Geilen CC, Ahmad W, Christiano A, Goerdt S, Orfanos CE (2000). Multiple familial cutaneous glomangioma: a pedigree of 4 generations and critical analysis of histologic and genetic differences of glomus tumors. J Am Acad Dermatol.

[19] Cohen PR (2022). Tender cutaneous neoplasms: case reports of patients with a symptomatic dermatofibroma and a new acrostic for painful tumors of the skin. Cureus.

[20] Najd Mazhar F, Shoushtarizadeh T, Mirzaei A (2018). Intravascular glomus tumor of the wrist causing chronic wrist pain. J Hand Microsurg.

